# Westminster Hospital

**Published:** 1829-12

**Authors:** 


### WESTMINSTER HOSPITAL.

### *Large Tumor in the Calf of the Leg.*

Amelia Beckett, æt. fifty-seven, admitted November 3d, 1829, with a very large tumor occupying the whole of the calf of the leg, and measuring in the centre seventeen inches and two eighths, being nearly three times the size of the other leg. She says that she was a stout healthy woman till within the last two years, when the tumor first made its appearance. She is now much emaciated, and has a very troublesome cough, accompanied by mucous expectoration. She first perceived a small lump in the left ham, which was very hard and painful, but did not, as far as she recollects, pulsate. When it was about the size of her double fist, a lancet was pushed into it, but nothing came out except a little blood. The tumor went on increasing, and her health gradually failing, till the present time. About a month after the opening of the tumor, she felt a tingling pain in the fore part of the sole of the foot, extending to her toes, which became so violent as to prevent her putting her foot to the ground. There was pain also extending from the outside of the leg, in the course of the fibular nerve, to the instep and anterior part of the foot. The skin over the calf of the leg is much on the stretch, and all the cutaneous veins are greatly enlarged. On the inside of the tibia, the tumor is softer than at any other part, colourless, and yielding an elastic springy feel, though not that of fluctuation; the same sensation is discovered at the back part, about where the heads of the gastrocnemius unite. No pulsation could be discovered by the ear, or by the stethoscope, unless when applied over two large arterial branches, which ran superficially over the back part of the gastrocnemius. There is general œdematous swelling of the leg and foot.

Mr. Guthrie observed that, although it resembled in some respects a diffused aneurism, still he did not believe it to be one; he considered it to be either a fungoid or anomalous tumor; and that, although he did not think amputation would save the woman's life, it was the only thing that offered her a chance. In this opinion Messrs. LYNN, CARLISLE, and WHITE, having fully concurred, the operation of amputation of the thigh was performed on Saturday, the 7th of November.

Mr. Guthrie said, before he commenced the operation, that he never applied the tourniquet when he had an able assistant to compress the artery against the pubes, (which Mr. HARDING had in the present instance undertaken,) inasmuch as it gave a great facility in the performance of the operation, saved a great deal of time, and was usually attended by less loss of blood than when the tourniquet was applied; and that he never used one unless he had bad assistants.

The thigh was amputated by Mr. Guthrie in *eighty seconds*, the first incision passing through the integuments and fascia, which allowed of their being retracted with the greatest facility, and without any dissection; a method of proceeding which, he said, he had first introduced, and which was of the greatest importance in regard to the saving of time and of pain to the patient. Two ligatures only were applied, one on the femoral artery, the other on the long descending branch of the external circumflex. Very little blood was lost. The woman bore the operation remarkably well.

On dissection of the limb, it was found that the tumor originated from the fascia covering the deep-seated muscles of the leg: it was cartilaginous and lardaceous to an inch and a half, or more, in thickness, containing within a matter partly pultaceous, partly of a curdy or cheesy texture; the pultaceous part being principally situated where the elastic feel was distinguishable previously to the operation. The posterior tibial and peroneal arteries were obliterated; the anterior tibial was given off at the usual place, and several large arteries ran down the back of the leg, external to the tumor. A cast of the dissected limb is preserved at the hospital.

The woman went on tolerably well for several days, and hopes were entertained of her recovery; but febrile symptoms and cough gradually increasing, she died exhausted on the 21st, fourteen days after the operation. Go to vol. lxiii. p. 128.

---

